# Candida dubliniensis candidemia in patients with chemotherapy-induced neutropenia and bone marrow transplantation.

**DOI:** 10.3201/eid0501.990119

**Published:** 1999

**Authors:** J F Meis, M Ruhnke, B E De Pauw, F C Odds, W Siegert, P E Verweij

**Affiliations:** University Hospital Sint Radboud, Nijmegen, The Netherlands. j.meis@mmb.azn.nl

## Abstract

The recently described species Candida dubliniensis has been recovered primarily from superficial oral candidiasis in HIV-infected patients. No clinically documented invasive infections were reported until now in this patient group or in other immunocompromised patients. We report three cases of candidemia due to this newly emerging Candida species in HIV-negative patients with chemotherapy-induced immunosuppression and bone marrow transplantation.


### *Candida dubliniensis* Candidemia in Patients with Chemotherapy-Induced Neutropenia and Bone Marrow Transplantation

**Jacques F. G. M. Meis,* Markus Ruhnke,† Ben E. De Pauw,* Frank C. Odds,‡ Wolfgang Siegert,† and Paul E. Verweij***
*University Hospital Sint Radboud, Nijmegen, The Netherlands;
†Humbold University, Berlin, Germany; and
‡Janssen Research Foundation, Beerse, Belgium

The recently described species *Candida dubliniensis* has been recovered primarily from superficial oral candidiasis in HIV-infected patients. No clinically documented invasive infections were reported until now in this patient group or in other immunocompromised patients. We report three cases of candidemia due to this newly emerging *Candida* species in HIV-negative patients with chemotherapy-induced immunosuppression and bone marrow transplantation.

Although *Candida albicans* remains the most common opportunistic yeast pathogen in patients with AIDS and other immunocompromised persons, species less susceptible to fluconazole are becoming more common (1). Recently, a newly described species, *Candida dubliniensis,* was isolated from oropharyngeal lesions in patients with AIDS living in Dublin, Ireland (2). *C. dubliniensis*, phenotypically very similar to *C. albicans* in producing both germ tubes and chlamydospores, has since been recovered from the oral washings of approximately 25% of 94 HIV-positive Irish patients with or without AIDS and 3% of 150 HIV-negative Irish persons (3,4), which suggests that this species belongs to the indigenous microflora of the oral cavity, albeit in a minority of healthy persons. Subsequent reports indicate that the species has a worldwide distribution (4). The role of *C. dubliniensis* as a pathogen has been limited to oral candidiasis. We now report three cases of candidemia due to *C. dubliniensis* in patients not infected with HIV. The yeasts were initially identified as *C. albicans* because each produced germ tubes and chlamydospores; this identification became suspect when equivocal carbohydrate assimilation patterns were obtained.

Address for correspondence: Jacques F.G.M. Meis, Department of Medical Microbiology (440), University Hospital Nijmegen, P.O. Box 9101, 6500 HB Nijmegen, The Netherlands; fax: 31-24-354-0216; e-mail: j.meis@mmb.azn.nl.

### Case 1

Graft-versus-host disease of the skin, liver, and digestive tract developed in a 39-year-old woman with chronic myelogenous leukemia after an allogeneic hematopoietic stem cell transplant in September 1995, during which she was treated with cyclosporine and high-dose prednisolone. Germ tube–producing *Candida* spp., later identified as *C. dubliniensis,* were isolated from stool samples obtained for routine testing. The white-cell count was 2.7 x $10^9$/L (72% granulocytes); 4 days later fever and ascites developed, and *C. dubliniensis* was cultured from three separate blood cultures (two sets obtained by venipuncture and one by the central venous line) taken on the same day (MIC fluconazole, 0.25 µg/ml). Ascitic fluid obtained by a sterile puncture also grew *C. dubliniensis*. Ascites was probably related to hypoalbuminemia. An echogram showed no radiologic evidence of liver candidiasis, although alkaline phosphatase was elevated (222 U/L; normal < 120 U/L). Treatment was started intravenously with fluconazole, 800 mg/day; 3 days later, *C. dubliniensis* were still recovered in one of five blood cultures taken over 2 days, but from then on, blood cultures were yeast-negative. Because the patient was in stable condition, the central line was not removed. Cytomegalovirus (CMV) disease also developed, which could explain the elevated alkaline phosphatase, with severe thrombopenia (<20 x $10^9$/L). The patient was given ganciclovir and hyperimmune gammaglobulin (Cytotect); *Staphylococcus epidermidis* bacteremia also developed, and the patient died 3 weeks after the onset of candidemia, with severe graft-versus-host disease stage IV, complicated by candidemia and CMV disease. Permission for autopsy was not granted.

### Case 2

In July 1995, a 5-year-old boy was treated with cytotoxic chemotherapy for relapsed nasopharyngeal rhabdomyosarcoma. Two episodes of bacteremia caused by *Streptococcus mitis* and *S. epidermidis* followed, and the boy was treated with ceftazidime and later ciprofloxacin (combined with vancomycin) for 3 weeks. Cultures of stools and oral specimens yielded germ tube–producing *Candida* spp. later identified as *C. dubliniensis*. Four days before the onset of candidemia the patient became febrile; *Staphylococcus aureus* and *C. dubliniensis* were cultured from sputum. At this time, the child was not aplastic (leukocyte count 2 x $10^9$/L). Flucloxacillin and ceftazidime were started. When fungal blood cultures were taken, the patient was very ill, had profuse diarrhea and high fever, and was leukopenic with a total leukocyte count of 0.3 x $10^9$/L (granulocytes <0.1 x $10^9$/L; thrombocytes 12 x $10^9$/L); 1 day later, three blood cultures, taken over 24 hours through a central line, yielded *C. dubliniensis* (MIC fluconazole, 0.5 µg/ml). Treatment with 12 mg/kg fluconazole was started immediately. *C. dubliniensis* were still being recovered from two blood cultures 2 days after treatment began, but after that, cultures remained sterile, and the patient gradually improved. The central line was removed 20 days after the last positive blood culture but was not submitted for culture. The patient was treated with fluconazole for 1 month (3 weeks intravenously, and 1 week orally) and was discharged 2 months after the onset of candidemia. No yeasts were recovered from fecal cultures and oral washes, but 1 month after discharge, oral washes again sporadically grew *C. dubliniensis*. The patient received further radiotherapy without any evidence of candidemia but died 1 year later of relapsed rhabdomyosarcoma.

### Case 3

An 8-year-old girl with sickle cell disease combined with ß-thalassemia and recurrent hemolytic crisis received an allogeneic hematopoietic stem cell transplant in January 1995. One year before, she had a splenectomy because of hypersplenism. The conditioning regimen for the transplant consisted of busulfan, cyclophosphamid, and antithymocyte globulin administered with a Hickman catheter. Cyclosporine and methotrexate were given as prophylaxis against graft-versus-host disease. A suspension of cotrimoxazole and amphotericin B was given as antiinfective prophylaxis. Seventeen days after transplant, sepsis syndrome and renal failure developed while the patient was still profoundly granulocytopenic (< 0.1 x $10^9$/L). Germ tube–producing *Candida* spp. later identified as *C. dubliniensis* were isolated from two sets of blood cultures drawn 6 hours apart from a peripheral vein and from two sets through the central venous catheter (MIC fluconazole, 0.25 µg/ml). The patient had no signs of oral candidiasis, and yeasts were not recovered from cultures (oral washes and stools). At this time the patient had already been treated for 72 hours with imipenem and vancomycin. Because of persistent fever unresponsive to broad-spectrum antibacterial agents, intravenous amphotericin B (30 mg) was empirically added. Once the results of the positive blood cultures became known, 5-flucytosin (100 mg/kg) was added to the regimen. After initiation of amphotericin B, later blood cultures remained negative for yeasts. The Hickman catheter was removed 14 days later when the patient had recovered from neutropenia. Catheter tip cultures remained negative. However, low grade fever persisted. Nonetheless, because the patient's condition was stable, treatment was changed to oral fluconazole (50 mg t.i.d.) for another 2 weeks and the patient was discharged. The cause of persistent fever was not identified, but approximately 6 months later, the patient recovered.

### Microbiologic Results

All yeast isolates were initially identified by germ tube and chlamydospore formation as *C. albicans*, but carbohydrate assimilation patterns by commercial test kits (Auxacolor,

Sanofi Pasteur, Paris and API 20C, Analytab Products Plainview, New York) gave equivocal results. Furthermore, the isolates did not elaborate ß-glucosidase, grew very weakly at 42°C, and failed to grow at 45°C (6); they produced dark green colonies on CHROMagar Candida plates (Becton Dickinson, Etten-Leur, The Netherlands) typical of *C. dubliniensis* (4,5) and abundant chlamydospores on rice-cream agar after 24 hours (2). In contrast with *C. albicans*, the yeasts isolated from our patients' specimens hybridized poorly with the *C. albicans*–specific Ca3 fingerprinting probe (5) and gave characteristic arbitrary primer phosphatase-polymerase chain reaction patterns for *C. dubliniensis* with primer RP02 (5'-GCGATCCCCA-3'). Each *C. dubliniensis* isolate yielded two major bands at 0.4 kb and 1.0 kb, with up to five weak bands ranging from 0.9 kb to 1.3 kb. In contrast, with *C. albicans*, the two major bands were never observed. Instead, each *C. albicans* isolate yielded approximately 15 bands of various intensity, ranging from 0.65 kb to 2.4 kb. Furthermore, banding patterns obtained with RP02 were clearly different from *C. glabrata*, *C. krusei*, *C. tropicalis*, and *C. parapsilosis*. In vitro susceptibility testing for fluconazole (powder provided by Pfizer BV, Capelle a/d IJssel, The Netherlands) was performed by the broth microdilution method with RPMI-1640 with L-glutamine, buffered with MOPS incubated at 35°C, and read after 48 hours according to NCCLS M-27A (7). *C. parapsilosis* ATCC 22019 and *C. krusei* ATCC 6258 were included as quality control strains. The isolates from patients 1 and 2 were deposited as CBS 8500 and CBS 8501 at the yeast division, Centraalbureau voor Schimmelcultures (CBS), Delft, The Netherlands.

*C. dubliniensis* was first described 3 years ago (2) and is genetically and phylogenetically distinct from *C. albicans* (8). Hitherto, its pathogenic role has been mainly restricted to oropharyngeal infections in HIV-infected persons and AIDS patients (3,5). In a recent study of *C. dubliniensis,* one isolate recovered from a blood culture and one from postmortem lung tissue was examined (6); however, no clinical data were described to allow determination of the pathogenic role. The cases we have described show that *C. dubliniensis* can cause candidemia in immunocompromised patients. However, these may not be the first cases of invasive disease due to this yeast. Identification and differentiation from other germ tube–producing yeasts on the basis of phenotypic characteristics has been problematic (8); therefore, the incidence and prevalence of this organism and its role in invasive disease have been difficult to determine. For instance, a strain of *C. stellatoidea* originally isolated in 1957 from the sputum of a patient with bronchopneumonia and deposited in the British Culture Collection of Pathogenic Fungi has been shown to be *C. dubliniensis* (2,3), and an isolate of *C. albicans* (from sputum of a Dutch patient) deposited in the culture collection of CBS in 1952 has been shown to be *C. dubliniensis* (Meis, unpub. obs.). In both cases, it has not been established whether the *C. dubliniensis* isolates were responsible for invasive infections.

Fluconazole appears to be less active against *C. dubliniensis* than against *C. albicans* (4) since *C. dubliniensis* is usually associated with recurrent episodes of candidiasis and protracted exposure to azole antifungal drugs in patients with AIDS. Fluconazole showed excellent in vitro activity against each of the *C. dubliniensis* isolated from the blood cultures of our patients; each patient responded well clinically. Nevertheless, it is too early to estimate the true susceptibility of this species to fluconazole. This requires the correct identification of the species, which now seems necessary, given its ability to cause invasive disease in patients treated for malignant diseases.

### Acknowledgments

We thank T. Rijs, L. Van Nuffel, and G. Dams for excellent technical assistance and J.P. Donnelly for discussion.

Dr. Meis is head of the Division of Bacteriology and Mycology, University of Nijmegen, The Netherlands, and consultant for medical microbiology and infectious diseases, University Hospital and Clinics. He is a member of the Nijmegen Mycological Research Group, and his research focuses on infections in immunocompromised patients, with particular interest in the management and diagnosis of invasive fungal infections.

### References

1. Abi-Said D, Anaissie E, Uzun O, Raad I, Pinzcowsli HM, Vartivarian S. The epidemiology of hematogenous candidiasis caused by different *Candida* species. Clin Infect Dis 1997;24:1122-8.

2. Sullivan DJ, Westerneng TJ, Hayes KA, Bennett DE, Coleman DC. *Candida dubliniensis* sp. nov.: phenotypic and molecular characterization of a novel species associated with oral candidosis in HIV-infected individuals. Microbiology 1995;141:1507-21.
3. Sullivan DJ, Coleman DC. *Candida dubliniensis*: characteristics and identification. J Clin Microbiol 1998;36:329-34.
4. Coleman DC, Sullivan DJ, Bennett DE, Morgan GP, Barry HJ, Shanley DB. Candidiasis: the emergence of a novel species, *Candida dubliniensis*. AIDS 1997;11:557-67.
5. Schoofs A, Odds FC, Colebunders R, Ieven M, Goossens H. Use of specialised isolation media for recognition and identification of *Candida dubliniensis* isolates from HIV infected patients. Eur J Clin Microbiol Infect Dis 1997;16:296-300.
6. Pinjon E, Sullivan D, Salkin I, Shanley D, Coleman D. Simple, inexpensive, reliable method for differentiation of *Candida dubliniensis* from *Candida albicans*. J Clin Microbiol 1998;36:2093-5.
7. National Committee for Clinical Laboratory Standards. Reference method for broth dilution antifungal susceptibility testing of yeasts. Approved standard M27-A. Wayne (PA): The Committee; 1997.
8. Sullivan D, Coleman D. *Candida dubliniensis*: an emerging opportunistic pathogen. Curr Top Med Mycol 1997;8:15-25.

